# Dante: A Psychological Study

**Published:** 1859-07-01

**Authors:** 


### Art. V.—DANTE: A PSYCHOLOGICAL STUDY.

The name of Dante is not only the most conspicuous in Mediæval history, but, as a poet, he takes rank among the foremost of any age or nation. In the literary firmament he shines "a bright particular star," and is one of the few master-spirits who have created the national poetry of their country, and given to its language and literature the impress of their own mind. Physically and intellectually considered, he must have been endowed with extraordinary powers; the mould in which he was cast was one of the choicest;—

> "The master mould of Nature's heavenly hand,
> Wherein are cast the heroic and the free,
> The beautiful, the brave."

Gifted with transcendent genius, he has been an object of the highest admiration to his countrymen for more than five centuries, and is regarded with increasing esteem and veneration throughout the civilized world.

The vigour and fertility of Dante's imagination, the faculty most essential to the poet, is displayed in the variety of characters and scenes which he has described, some remarkable for their beauty and pathos, and others for their terrible grandeur and sublimity. His delineation of Capaneus, unsoftened by the eternal fire, and obdurate as ever, scarcely yields to the *Prometheus* of Æschylus, and is the prototype of the Satan of *Paradise Lost.* Dante tells us that he asked Virgil,—

"Who is that mighty one, morose and grim,
Who careless of the burning seems to lie,
So that the fire-shower cannot soften him?
And he, as to my leader I apply,
Perceiving 'twas of him I thus enquire,
Cried, 'What I was alive, such dead am I.
If incensed Jupiter his workman tire,
From whom he snatch'd the thunderbolts that day,
Which was my last, and struck me in his ire;
If he—the rest all spent by turns while they
The sledge in Mongibello's black forge wield—
Cry, 'Help, good Vulcan, help!' as in the fray
He cried of old in the Phlegræan field,
And launch his bolts at me with all his might,
A joyful vengeance it shall never yield."

*Inferno*, Canto xiv. 46.*

In sublimity he is only surpassed by the Hebrew prophets, by Homer, and by our own Milton; yet even in the most thrilling and tremendous descriptions of infernal misery, we are ever and anon presented with images of beauty and calm delight, which are all the more pleasing and welcome from their contrast with the scenes of suffering, the timeless gloom, and the air for ever shaken, from which we have just escaped, and into which we have so soon again to pass. It is as if when treading "over the burning marle," we suddenly came upon some happy valley, or entered some sylvan shade, where the song of birds is heard amidst the foliage, or the music of the rill that murmurs on the verge of the enamelled green. Take for instance the limbo of the unbaptized, to which we shall again refer; or the tale of Ser Adamo:—

"O ye who even in this world accurst,
I know not wherefore, no affliction have,'
He thus began, 'Behold and hear rehearsed

---

* Our quotations are from a new translation which has just made its appearance, entitled, "*The Trilogy; or, Dante's Three Visions. Inferno, or the Vision of Hell.* Translated into English in the Metre and Triple Rhyme of the Original; with Notes and Illustrations. By the Rev. John Wesley Thomas. Henry E. Bohn, York-street, Covent-garden, 1859."

We would commend this translation to all lovers of Dante for its beauty and accuracy. It deserves, moreover, especial notice from its being executed in the rhyme of the original. The author has indeed successfully overcome the great difficulty experienced by all translators of the immortal Florentine's poem, and at which the majority have blenched, to wit, the preservation of the metre and triple rhyme of the original, while at the same time rendering truthfully the poet's meaning. In the copious *Notes* also, there is much interesting and varied information, and a great clearing up of obscure and disputed passages:

What Ser Adamo suffers in this cave.
  Alive I had enough of all at will,
  And now, alas! one drop of water crave.
The brooks which downward o'er each verdant hill
  Of Casentino to the Arno flow,
  Making their channels fresh and soft, are still
Always before mine eyes, nor vainly so.
  For more their image dries me up than this
  Disease which in my unflesh'd cheeks I show.
Now from the place in which I did amiss,
  Derives avenging justice a supply
  Of means to augment my sighs in hell's abyss.
There, in Romena, did I falsify,
  The coin that bears the Baptist on its front,
  For which I left my body burnt on high.
But could I see tormented here the Count
  Guido, or Alessandro, or their brother,
  I would not change the sight for Branda's fount."

*Inferno*, Canto xxx. 61.

But while the *Divina Commedia* presents us with such admirable specimens of vigorous description, it also excels in portraying the deep feelings of the mysterious human heart. What Piero Della Vigne did for his imperial master, Frederick II., Dante does for his readers :—

"I then am he who once held both the keys
  Of Frederick's heart, and who in that high post,
  Opening and shutting turn'd them with such ease
None else his secret confidence could boast."

*Inferno*, Canto xiii. 58.

This mastery over the passions is shown alike in the despair which petrifies Ugolino, as the wretched father beholds his children droop and die with famine ; in the self-devotion of Francesca and her love, unquenched by misery and death ; in the melting influence of the sound which makes the new pilgrim of love start, when the evening bell, swinging in the far-off tower, tolls the knell of the dying day ; in the blasphemies of the lost on the shores of Acheron ; in the milder sorrows of the repentant in Purgatory ; and in the joy with which the poet hails the object of his undying attachment, Beatrice, in the realm of blessedness. Dante was, moreover, in a remarkable degree the poet of his time ; he has portrayed the creed, philosophy, politics, and superstition of the age in which he lived. His conversation with Farinata in the tenth Canto of the *Inferno*, says Mr Hallam, "is very fine, and illustrative of Florentine history." That with Piero Della Vigne, in the thirteenth Canto, exhibits in a light equally striking the cabals which infested the court of the Emperor Frederick II. ; while the narrative given of himself by Guido Montefeltro is a damning exposure of the Papal Court and its intrigues and tyranny, under the ambitious and unprincipled Boniface VIII., "the Prince of the new Pharisees." Dante's writings afford also an illustrious example of self-portraiture. In *La Vita Nuova*, the earliest of his known productions, he paints the workings of that youthful passion which helped to stamp his destiny as a poet, and inspired his hymn of the eternal rest. But in his great poem, the *Divina Commedia*, he presents us with a mirror which reflects the mind and character of the author, as well as his life and times. For with all the fire and sublimity of his genius, his personal feelings and experience form the groundwork of his poem, and furnish its most glowing materials.

His education is referred to in his conversation with Brunetto Latini, whom in hell he recognises as his old preceptor, and says:—

> "For in my memory fix'd now grieves my heart,
> The dear and good paternal image known,
> Of you on earth, where with a master's art,
> You taught me how eternity is won.
> How dear I hold the lesson, while I live
> 'Tis fit should by my eloquence be shown."
>
> *Inferno*, Canto xv. 82.

In the very commencement of the poem, he describes, though in a strain somewhat allegorical, his thoughts and feelings in middle age, when he called himself to account for his previous errors, and resolves to enter on a new life. Canto i. 1. His exile is foretold by Farinata, Virgil and Vanni Fucci, and his misfortunes and famé are foreshown by Brunetto Latini and Beatrice.

His power of sarcasm and invective was terrible; witness his imprecation on Pisa for its heartless cruelty to the innocent children of Ugolino, his reproof of the Emperor Albert for permitting the continuance of Italian anarchy, and the reproach with which he thunder-strikes Pope Nicholas IV. and the Simonists in hell. We quote the first and last of these examples:—

> "Ah, Pisa! shame of all who appertain
> To that fair land with language of soft sound,
> To punish thee since neighbours yet abstain,
> Capraia and Gorgona from the ground
> Rise, and a mole o'er Arno's entrance throw,
> Till with her waters all in thee be drowned.
> That he thy castles had betray'd, although
> Count Ugolino was accused by fame,
> His children thou shouldst not have tortured so.
> The shield of innocence which youth may claim
> (New Thebes!) Uguccion and Brigata share."
>
> *Inferno*, Canto xxxiii.

In relating his conversation with Pope Nicholas IV., he says:—

"I know not if too rashly I my mind
    Express'd, but my reply this burden bore;
    'Alas! now tell me, when our Lord inclined,
To put the keys into St. Peter's power,
    What treasures did he first of him demand?
    None:—'Follow me,' he said, and asked no more.
Peter and th' others of Matthias' hand
    Nor gold nor silver took, when lots they cast,
    For one in Judas' forfeit place to stand.
Then stay, where thy just punishment thou hast.
    And look that thou guard well that wealth ill-gain'd,
    Whence thou against King Charles embolden'd wast.
And if it were not that I am restrain'd
    By reverence for the keys which once did fill
    Thy grasp, while cheerful life to thee remain'd,
The words I speak would be severer still,
    Because your avarice the whole world hath grieved,
    Trampling the good and raising up the ill.
You shepherds the Evangelist perceived,
    When her who on the waters sits he saw,
    And who with kings in filthy whoredom lived.
Her who with seven heads born could also draw
    From the ten horns conclusive argument,
    While yet she pleased her spouse with virtue's law.
What could the idolater do more who bent
    To gold and silver, which you make your god?
    But to a hundred worship ye present,
For one! Ah, Constantine, what ills have flow'd
    Though not from thy conversion, from the dower,
    Which to thy gift the first rich father owed."

*Inferno*, Canto xix. 88.

The great characteristics of Dante are his earnestness, energy, and elevation of sentiment, for which his compressed diction, and the emphatic cadences of his metre furnish an admirably suitable vehicle. Of Dante's pithy and pungent style, Cary's blank verse may enable the reader to form some notion, but can give no idea of his music—that melody with which he wins and pleases the ear, and charms the imagination, while it indelibly impresses on our memory the lessons of truth and wisdom which it is his object to impart. It must indeed be admitted that the *Divina Commedia* is not without its faults—for what human work is perfect? "Aliquando dormitat Homerus." And the instances in which Homer has been observed occasionally to nod, are in the management of his machinery, or treatment of the gods of the Olympus. The blending of Pagan mythology with Christian tradition and the truths derived from Holy Scripture, makes

Dante's poem in some parts appear like the debateable ground between the ancient Superstition and the newer Faith; in which, however, the latter is victorious, and the dethroned and desecrated gods of the Pantheon, transformed to demons, are dragged at the chariot-wheels of the conqueror. But if in this respect the poet falls short of that higher and purer standard of taste which has been recognised in later times, this defect has been amply compensated, not only by his innumerable beauties, but especially by the high purpose with which he wrote—a purpose hitherto unaccomplished, indeed, but not less important at the present moment than when the *Divina Commedia* came fresh from his hands and from the depths of his soul. To use the language of an eloquent and learned writer in the *Athenæum*—

"He desired the regeneration of his country, and her restoration to that place and power among the nations of Europe, from which internal dissensions and strife, the madness of opposing factions, and the selfishness of oppressive and tyrannical rulers, had deposed and degraded her. Poet, patriot, philosopher, historian, Dante Alighieri shines forth a great and glorious light in the stormy firmament of the Middle Ages; and in the midst of wild confusion and the crash of elements, rises up the zealous advocate of order, stability, and the supreme divine rule."

To ascertain the circumstances which contributed to mould, and fashion, and direct the genius of Dante, an extensive acquaintance with the age in which he lived, and of that which immediately preceded it, as well as with his ancestry, birth, education, and condition through life, is necessary. As far as our limits will admit, we shall exhibit these in their immediate bearing on the subject of this article.

Dante was born in the thirteenth century, and arrived at manhood when Florence, his native city, was foremost in civilization, commerce, arts, and freedom. He was nobly descended; his great-grandfather, Cacciaguida Elisei, having accompanied Conrad III. in his crusade to the Holy Land, was knighted by that emperor, and died on the field of battle, A.D. 1147. In the *Paradiso* he relates his adventures to Dante, with an interesting account of Florence and the manners of her citizens, before the breaking out of the great feud between the Guelfs and Ghibelines. Dante, while yet a child, was deprived of his father, but his education was amply provided for by the care of his mother, who had been left in affluent circumstances, and who obtained for her son the best instructors that Florence could supply. The early indications of his genius appeared in a noble and contemplative disposition, and that enthusiastic love of learning and study which is the surest presage of distinction, and which accompanied him through every period of his life. Among his intimate friends were some of the most celebrated men of his time—philosophers, poets, musicians, and painters; and in the pursuit of wisdom, he not only studied in the famous universities of Padua and Bologna, but is also said to have visited those of Paris and Oxford. The vicissitudes of his life were great. Although not a warrior by profession, he was in the battle of Campaldino (*Inferno,* xxii. 5), and in the victory there achieved by his countrymen over the Ghibelines of Arezzo, distinguished himself by his bravery. In the thirty-second year of his age, he entered on the honours and anxieties of the chief-magistracy in his native city; but soon after was banished with his party, the Bianchi (Whites), and afterwards in his absence, by a most iniquitous sentence, and without a hearing or trial, condemned to be burnt alive!

In Dante's age, and long after, the authority of Aristotle was undisputed and supreme in the European schools of learning. How great was Dante's veneration for the Stagyrite, may be seen in the epithet by which he distinguishes him, "Il maestro di color che sanno" ("The master of those who know"), *Inferno,* iv. 131; and also in the somewhat prodigal and ostentatious display which he makes of the Aristotelian lore in the eleventh canto of the *Inferno,* where he formally quotes the *Physics* and the *Ethics,* and gives a complete synopsis of the sins and crimes which have been or can be committed, according to their various degrees of demerit, according to "the Master," whose authority was then paramount; so that "*Ipse dixit,*" with a quotation from his works, was deemed a sufficient and all-conclusive argument in any controversy. This may seem almost incredible to us; yet it is not less certain, that even in Theological speculations, though himself a heathen, his authority was appealed to by Christian divines. Dante consults him in arranging the different degrees of suffering in the world below; and Melanchthon complains, that even in his time, that sage's works were read in some churches instead of the Gospels. In the schools they were both law and gospel; but it is to the Reformation that we are indebted for the emancipation of the human understanding from the fetters of human authority.

In the age of Dante, the Ptolemaic system of the universe was equally prevalent; the poet accordingly regards the earth as immovably fixed in the centre, the sun and all the planets, as well as the fixed stars, moving round it once in every twenty-four hours.* Nor need we marvel much at this, when we consider that three hundred years later, Milton, who had conversed with Galileo, although inclining to the theory which that philosopher taught, speaks doubtfully of the comparative merits of the old and new systems of astronomy, and has often adapted his expres-

* See an original, ingenious, and beautifully-coloured diagram, illustrative of Dante and the Ptolemaic system, prefixed as a frontispiece to Mr. Thomas's *Trilogy* of Dante.

sions and ideas to the Ptolemaic theory rather than to the Copernican system.

Dante's fame as a poet should not make us forgetful of his claim to be ranked among the metaphysicians of his age. He everywhere exalts and glorifies the intellectual nature and greatness of man, while glorifying the Supreme. Thus he says—

"It is the intention of God that every created thing should represent the Divine likeness, as far as its nature allows, according to the saying, 'Let us make man in our own image.' And though it cannot be said that inferior natures are made in the image of God, still, all may be said to bear a similitude to Him, since the whole universe is nothing else but a trace of the divine goodness."—*De Monarchiâ.*

"The best state of man is that in which he is most free . . . . and the foundation of our liberty is the freedom of the will, which many talk about, but few understand. And this liberty is the greatest blessing which God has bestowed on human nature, since by means thereof is secured our happiness here and hereafter."—*ib.*

"Everything desires its own perfection, and in this all its desire will rest, and it is only for its sake that everything else is desired; and it is this desire that seems to make every pleasure deficient, since there is no happiness in this life so great as to allay this thirst and banish it from the mind."—*Convito.*

"When one speaks of a man's living, it is implied that he employs his reason, which is his special life, and the use of his noblest part."—*ib.*

"It is manifest that universal peace is of all things best suited to the promotion of human happiness. . . . . Hence the voice from heaven spoke not of riches, nor of honours, nor of beauty, but of peace. For the heavenly host cried, 'Glory to God in the highest, and on earth peace, goodwill towards all men."—*De Monarchiâ.*

The influence of the classical writers of Greece and Rome on the mind of Dante is everywhere discoverable—in the deference which he pays to Virgil as his teacher, guide, and master; in the veneration with which he regards the shades of Homer, Lucan, Ovid and Horace (*Inferno,* Canto iv. 88 &c.); in his frequent adoption of the Greek mythology; and, above all, in his having contrived—for the express accommodation of the great classical heroes, poets, and philosophers of antiquity, whom the orthodox theology of the time had excluded from heaven—a kind of paradise in hell.

But lest any reader should doubt our correctness in the last-named instance, or think that we misinterpret the poet's meaning in ascribing to him this "large economy," we quote the passage:—

"Now to a noble castle's foot we came,
Seven times with lofty walls encompass'd round;
And round it also flow'd a pleasant stream,
O'er which we pass'd as if upon firm ground:
Through seven gates entering with the sages there,

We reach'd a meadow with fresh verdure crown'd;
With grave slow eyes, the crowds assembled there
In their appearance of great majesty;
And as they talk'd their words were sweet and rare.
Thus to one side retiring enter'd we
An open place, light, lofty, and serene;
So that all there were visible to me.
There just above, upon the enamell'd green,
The mighty spirits I could recognise,
Whom I esteem it honour to have seen."

*Inferno*, Canto iv. 106, &c.

The remainder of the Canto contains a description of them by name.

Besides the descent of Ulysses into Hades related by Homer in the *Odyssey*, and that of Æneas, by Virgil in the *Æneid*, many visions of the other world had been related and proclaimed by the Monkish writers during several centuries preceding the age of Dante. The exhibition of mysteries, or dramas on sacred subjects, in churches and elsewhere, were the earliest scenic performances in Europe after the downfal of the Roman empire: and these were still more likely to excite the imagination of Dante and direct him in the choice of his great subject. Thus the operas, or musical dramas, which, in a later age, our own great poet witnessed when in Italy, are supposed to have influenced the mind of Milton in the conception and composition of *Paradise Lost.** A remarkable instance, and possibly one of the earliest of those mediæval dramatic performances, occurred in the lifetime of Dante and in his native city. On the occasion of a public festival, under the auspices of the clergy, to celebrate the entrance of the papal legate, a representation of infernal torments was exhibited in the bed of the Arno, which was converted into the gulf of perdition, where all the horrors invented by the prolific imagination of the monks were concentrated. But in the midst of this extraordinary drama, the wooden bridge on which the multitude of spectators were congregated gave way beneath them, and the shrieks and groans of simulated sufferers were suddenly exchanged for those of real ones. This catastrophe, although it happened about two years after Dante's exile had commenced, must, when reported to him, have made a deep and indelible impression on his mind. It is one calamity among others which the poet is supposed to allude to, by a kind of *ex post facto* prognostication. *Inferno,* Canto xxvi. 9.

* In Todd's notes to Milton may be seen the passages from the Italian opera which are supposed to contain the *germ* of his great poem. It has been said that Satan's address to the Sun, "O thou that with surpassing glory crowned," &c., was at first intended as the commencement of an English opera. The Italian opera was not introduced among us till the beginning of the eighteenth century. See *Tatler*, No. 4; and *Spectator*, Nos. 5 and 18.

The genius of Dante cannot but have been greatly influenced and directed by the early poetry of France. The Troubadours, who employed the Provençal tongue, were the instructors of Europe in the rules of modern versification. They visited every court; their presence was welcomed by kings and nobles; and all the historians of Italy have recognised their powerful influence on the literature of that country. The first lispings of the Italian muse were but humble imitations of Provençal lyrics, and it was from among these, which in their day had been so famous, that Dante arose to pale their ineffectual beams by the superior splendour of his genius. But besides the Troubadours, whose genius was lyric, and who sung of "faithful loves," there were the Trouveres of Northern France, whose genius was epic, and who in the Wallon dialect sung "fierce warres." The compositions in which they celebrated the exploits of Arthur and the Knights of the Round Table, and those of Charlemagne and his Paladins, were not less popular in Italy than those of their Provençal brethren. Dante describes Paolo and Francesca di Rimini as reading for their amusement the Romance of *Lancelot du Lac*, which was commenced by Christian de Troyes, and completed by Godfrey de Ligny (*Inferno*, Canto v. 128). He alludes to the same romance, Canto xxxii. 62, and *Paradiso*, Canto xvi. 15. And besides these allusions to the romances of the Trouveres, their spirit may be recognised in the majestic allegories of Dante, who, according to Sismondi, has taken for his model the most celebrated and most ancient of them, the *Romance of the Rose*, which, however, he has infinitely surpassed.

The two passions which predominated in the breast of Dante were love and patriotism. We all know that early attachments are often the purest, and the most lasting in their influence; and how some object of our boyish passion, whom death or distance has separated from us, continues to exist for us, enshrined in our memory, or visiting our dreams, as when she rose upon us like an Eve, or as when we last saw her, long years ago, in all her virgin charms. Thus it was with Dante. In the ninth year of his age he first saw a young lady a few months older than himself, an event which made on him an indelible impression. The vision of Beatrice Portarini, which at a festival given by her father to the young people of the city, on May-day, 1274, never departed from him; but awakened in his bosom a feeling which, intensified by that glowing clime and the fires of his own genius, became his ruling passion. As in the case of another great poet of our own country, the object of his first and passionate love could not be his. Yet

"—— She was his life,
The ocean to the river of his thoughts."

After several years of declining health, she died at the age of twenty-five;—unconscious, probably, or but half conscious, of that admiration with which she had inspired the youthful poet. But of the extent of its influence on his mind and character, every reader of Dante cannot but be aware. To her he consecrated the earliest strains of his lyre; and in his maturer age, while he is passing through the regions of blessedness, listening to celestial harmony, amidst the shining companies of saints and angels, *her* presence heightens heaven.

Equally intense was his love of native land, and the bitter disappointment of perpetual exile from it. Throughout the *Divina Commedia* we see the banished magistrate of Florence, the exiled statesman, whose bowels yearn to be restored to his native country.

"La carità del natio loro mi strinse."

For the love which he bears to Florence he stoops to gather up and reverently deposit the human spoils of one of her citizens whom he meets with in the hell of suicides. And yet how terrible are his denunciations against Florence for the cruelty and crimes of her children. In Canto xvi. of the *Inferno*, Jacopo Rusticucci, a Florentine, asks Dante,

"If still as wont reside
Courtesy and valour in our urban state,
Or if thrust forth by all they wander wide?"

To which Dante replies:—

"The new race and the sudden gains in thee,
O Florence, have produced excess and pride,
For which even now thou weepest wofully."
L. 67.

And in the commencement of Canto xxvi. we have the following apostrophe:—

"Florence exult! thy greatness who can tell;
O'er sea and land thy rushing wings resound;
Meantime thy name hath spread itself through hell.
Five such among the plunderers there I found
Thy citizens, whence shame befalleth me,
And to thyself no glory can resound.
But if our dreams near dawn may claim to be
The truth, much time will not elapse ere thou
Feel what, not Prato only wisheth thee;
And 'twould not be untimely if 'twere now.
Would that it were so, since it must take place;
'Twill grieve me more the more with age I bow."

Again, how touching are his appeals, and how unceasing and unwearied were his endeavours to obtain a revocation of his sentence. Yet how high principled he was, appears from his refusal to accept even restoration to his country and patrimony, on the condition of acknowledging his fault and asking forgiveness. "No, father," he exclaims, "it is not this way that shall lead me back to my country. But I shall return with hasty steps, if you or any other can open to me a way that shall not derogate from the honour and fame of Dante." It is impossible to withhold our admiration from so high-souled a sufferer. He refused to demean himself even to escape the privations of poverty, the bitterness of dependence on strangers, and the anguish of irrevocable exile. The memory of the wrongs he had endured was indelible, yet amidst all his eloquent appeals and denunciations, we recognise, throughout his life-long struggle, an ardent love to his ungrateful country which refused to turn itself to hatred.

The anti-papal spirit of Dante is a remarkable fact in his history. The fact is not dependent for its proof on reasoning so subtle and recondite as that of Rosetti, who insists that the *Divina Commedia* is an allegory, the sense of which is figurative and esoteric. Dante, for aught we know, *may* have belonged to a secret society possessing a system of signs and an enigmatical language. But if its great secret was hostility to the papacy, that secret has been very *ill-kept* by him, and he ought to have been dismembered for betraying it! We believe Dante to have been too bold and plain-spoken to have required such a method. His hostility to the papacy is patent and undeniable, and is the more remarkable inasmuch as he preceded Wickliffe by some years, and Luther by two centuries. In the twelfth and thirteenth centuries there were numerous sects in France, Brabant, and even in Italy, who were opposed to the doctrines and practices of the dominant church, and who were consequently the objects of papal vengeance; and almost every government then existing lent a willing hand towards crushing them. A crusade against those in the south of France was proclaimed by Innocent III. in the beginning of the thirteenth century, and pardons and paradise (besides plunder) were liberally promised by that pontiff to all who should assist in that holy war. A similar crusade was proclaimed by Clement V. in 1305, against Fra Dolcino and his followers in Italy. The heroism and fate of this leader of a sect, which were worthy of either the tragic or the epic muse have been commemorated by Dante. Mohammed, addressing Dante, says:—

"Thou who perchance the sun mayst shortly see,
To friar Dolcin then this warning bear,
If here he would not soon my follower be,
That corn be stored, lest snow besiege him there,
And victory to the Novarese convey,
Which else for them no light achievement were."
*Inferno*, Canto xxviii. 58.

L. Mariotti, in his *Historical Memoir of Fra Dolcino and his Times*, regards the above as a hint from Dante to one with whose object he sympathized, and whom he might "almost contemplate joining." We rather think it an allusion penned after the fate of Dolcino had been decided, though in the form of a warning and put into the mouth of Mohammed, who is supposed to utter it some few years previously to its fulfilment—namely, at the date of the vision in 1300. Dolcino needed no such caution, which, if given, would in the circumstances have been useless; the Bishop of Vercelli, who commanded the army of the faith, having desolated the whole country round, and removed its inhabitants, for the purpose of starving the heretics whom he could neither conquer by carnal nor spiritual weapons. Dolcino and his little band in their mountain fastness withstood the forces of the crusaders for two years, and whether assailed or assailing were victorious on every occasion. But the heretics were at length vanquished by famine, and then burnt at Vercelli, June 1st, 1307; Dolcino as their leader having first been torn with red-hot pincers. Although the only accounts of him which have come down to us are the writings of his enemies, yet it is admitted that he possessed extraordinary talents as a popular orator and a military strategist, and that both he and "Sister Margaret" endured their fate with a courage and firmness worthy of a better cause.

But besides the sects which lay under the ban of the Papacy, and were the objects of its relentless persecution, there were many among the leading minds of Italy, who although continuing in outward communion with the Church of Rome, had received no small portion of evangelical truth; while the spirit of inquiry, and the impulse given to thought by the revival of classical learning, had tended to open their eyes to the corruptions of the Church, and the daring usurpations of its chief. Dante's politics were very nearly, if not quite, identical with those of Dolcino and his followers. Gregory VII. had reared up the Papal authority on the ruins of the civil power; it was for an Emperor to reassert his supremacy, and redress the grievances of Italy by the humiliation of the Papacy. The Abbot Joachim had foretold as much; and his predictions were immensely popular. He is eulogized by Dante, who places him among the saints in Paradise.

> "Il Calvarese abate Giovacchino
> Di spirito profetico dotato."
>
> "The abbot of Calabria Joachim,
> Who with prophetic spirit was endow'd."
>
> *Paradiso*, Canto xii. 140.

Dante's treatise *De Monarchia* was written to prove the independence of the civil magistrate; and is worthy of perusal for the strength and freedom of its arguments. It is therefore not surprising that, as the only method of answering them with which he was acquainted, Pope John XXII. had it publicly burnt a few years after Dante's death, and that it has found a place in the Roman catalogue of heretical and prohibited books.

Dante, without question, like Luther at the commencement of his career, acknowledged the spiritual supremacy of the Pope and held most of the doctrines of the Church of Rome. But in early life he had become acquainted with the Holy Scriptures, and the result is obvious throughout his great poem. To them he ascribes the light of truth which had been poured into his soul, and not to his own labours, learning, experience, or philosophy. In reply to the question, "What is faith?" he answers in the words of St. Paul, "Faith is the substance of things hoped for, the proof of things not seen." St. Peter then says, "This precious jewel, whence comes it to thee?" Dante replies, "The copious rain of the Holy Spirit, which is poured out on the Old and New Testament, and an argument which so conclusively convinces me that every other proof seems obtuse in comparison of it." In another part of the dialogue he says, "I believe in one God, sole and eternal, who, unmoved himself, moves all heaven with love and desire. And for such belief I have not only physical and metaphysical proof, but also the truth which is showered down from heaven bestows it on me, through Moses, the prophets, the Psalms, the Gospel, and through you who wrote when the burning spirit made you sublime. And I believe in Three Eternal Persons, and these I believe in essence one, so one and so trine that they admit conjointly of *are* and *is*. Of this profound and divine condition, which now I merely touch, the evangelic doctrine (*l'evangelica dottrina*) hath often sealed the impress on my mind. This is the principle, this is the spark, which afterwards is kindled into a vivid flame, and shines in me like a star in heaven."—*Paradiso*, Canto xxiv. 52—147.

Dante appears to have been endowed with a rare sagacity. His mind went far ahead of the times in which he lived. In the imaginary voyage of Ulysses to the Antipodes, in Canto xxvi. of the *Inferno*, he has foreshadowed the discoveries of the Portuguese in the southern hemisphere, and may have given a hint to Columbus himself.

Ulysses, in describing his voyage southwards, says—

"Each star of the other pole, as on we bore,
The night beheld, and ours had sunk so low,
That now it rose not on the ocean-floor."

*Inferno*, xxvi. 127-9.

And relating his own voyage to the Mount of Purgatory, Dante says:—

"I turn'd me to the right, and fix'd my mind
On the other pole, and those four stars I saw,
Ne'er seen save by the earliest of mankind."
*Purgatorio*, i. 22-4.

The southern cross, which Dante here describes, consists of four stars. Amerigo Vespuccio, in his third voyage in 1501, first applied these lines of Dante to that magnificent constellation, which is to the southern what our pole-star in *Ursa Minor* is to the northern hemisphere.

We have Cuvier's theory of the earth anticipated in a single line of the *Inferno*, Canto xii. 43:—

"The world has oft been into chaos turn'd."

One can hardly withhold from Dante the credit of having been acquainted with the state of the pre-Adamite earth, and with its enormous occupants, now extinct, and so recently unveiled by geology. Of "the horrible giants," he says:—

"Nature, indeed, when she declined the art
Of forming such as these, did what was meet,
Taking from war these vassals grim and swart;
And if the elephant and whale so great
Repent her not, who ponders as he ought
Holds her herein more just and more discreet."
*Inferno*, Canto xxxi. 49.

In the thirty-fourth canto, he displays his knowledge of gravitation, and the sphericity of the earth, when he speaks of its centre as the place

"Towards which all heavy things from all parts tend."
Line 111.

Dante has been described as of middle stature and grave deportment, rather long-featured, with large eyes, aquiline nose, prominent cheekbones, dark hair, and an under lip that slightly pouted. Besides the description given by contemporary writers, we have the portraiture of his calm, dignified, and interesting features from the pencil of his friend Giotto, the gifted pupil of Cimabue. It is said that Dante had somewhat impaired his sight by his intense application to books and study. His dress was plain, and his habits temperate. He was at times a little absent and abstracted, as well as a little sarcastic. G. Villani, the historian of Florence, says, that "upon the strength of his knowledge he was somewhat haughty and disdainful, and like an ungracious philosopher could not endure to converse with the ignorant." It is probable that this made him enemies, and increased the griefs and troubles with which he was beset. As an instance of biting sarcasm, it is related that while Dante was the guest of San Francisco Scaglieri, surnamed *Il Grande* (The Great), one day when that nobleman was amusing himself by listening to the court jester or fool, he asked the poet why it was that so many of the nobles had a much greater regard for the fool than for him? To which Dante replied, "Because they are by nature much more like him than me, and therefore they naturally prefer his society to mine."

Dante was not only a man of vast energy, but he also seeks to inspire his readers with the same temper. His disdain of those who trifle with great affairs, or give way to useless repining, or waste their lives in indolence, was unbounded. He speaks of them as "drones who never truly lived," as "abject wretches whom God and godless men alike must hate:" and he gives them a place with the neutral angels, for ever hovering in the outskirts of damnation. And among them he places the hermit Pope, Morone, Celestine V., for having, when raised to the Papal throne, instead of exerting himself vigorously to reform the Church, sighed for his retirement, and at the instance of the crafty Cardinal Cajetan (afterwards Boniface VIII.), abdicated his office, and thus missed the opportunity of effecting that great work which had been generally expected from him. This Dante calls *il gran rifiúto*, "the great refusal." And what a lesson have we in the doom of the discontented!—

"'Fix'd in the mire,'
They cry, 'we once were sad in the sweet air
Which the bright sun makes gladsome with his beams,
Carrying the sluggish smoke within us there:
Now are we vex'd in these black muddy streams.'"
*Inferno*, Canto vii. 120.

It is pleasing to observe the increased and increasing interest in the character and writings of Dante which is felt in our own country, as it tends not only to increase our sympathy with Italy in her sorrows and sufferings, and our indignation at her wrongs, but also affords us a salutary warning against the indulgence of those passions and vices by which Italy was first divided, and then made the prey of domestic and foreign tyrants. We would fain see Italy freed from her oppression and her woes. We hope to see her self-reliant, united, free, and happy. And that she may be so, let her children listen to the teachings and admonitions of their own poet—of him whose name still gives renown to the land which his genius illumined, and who has pointed out so clearly the causes of Italian degradation and bondage: and let them steadily pursue that path which he has indicated as the only way to national greatness and prosperity, and thus prove themselves, at length, worthy of their illustrious countryman and predecessor, Dante Alighieri.

